# Role of superoxide–nitric oxide interactions in the accelerated age-related loss of muscle mass in mice lacking Cu,Zn superoxide dismutase

**DOI:** 10.1111/j.1474-9726.2011.00709.x

**Published:** 2011-05-06

**Authors:** Giorgos K Sakellariou, Deborah Pye, Aphrodite Vasilaki, Lea Zibrik, Jesus Palomero, Tabitha Kabayo, Francis McArdle, Holly Van Remmen, Arlan Richardson, James G Tidball, Anne McArdle, Malcolm J Jackson

**Affiliations:** 1 Institute of Ageing and Chronic Disease, University of LiverpoolLiverpool L693GA, UK; 2 The Barshop Institute for Longevity and Aging Studies, University of Texas Health Center at San AntonioSan Antonio, TX 78229-3900, USA; 3 Molecular, Cellular and Integrative Physiology Program, University of California at Los AngelesLos Angeles, CA 90095-1606, USA

**Keywords:** accelerated aging, aging, reactive oxygen, species, skeletal muscle

## Abstract

Mice lacking Cu,Zn superoxide dismutase (SOD1) show accelerated, age-related loss of muscle mass. Lack of SOD1 may lead to increased superoxide, reduced nitric oxide (NO), and increased peroxynitrite, each of which could initiate muscle fiber loss. Single muscle fibers from *flexor digitorum brevis* of wild-type (WT) and *Sod1*^*−/−*^ mice were loaded with NO-sensitive (4-amino-5-methylamino-2′,7′-difluorofluorescein diacetate, DAF-FM) and superoxide-sensitive (dihydroethidium, DHE) probes. *Gastrocnemius* muscles were analyzed for SOD enzymes, nitric oxide synthases (NOS), and 3-nitrotyrosine (3-NT) content. A lack of SOD1 did not increase superoxide availability at rest because no increase in ethidium or 2-hydroxyethidium (2-HE) formation from DHE was seen in fibers from *Sod1*^*−/−*^ mice compared with those from WT mice. Fibers from *Sod1*^*−/−*^ mice had decreased NO availability (decreased DAF-FM fluorescence), increased 3-NT in muscle proteins indicating increased peroxynitrite formation and increased content of peroxiredoxin V (a peroxynitrite reductase), compared with WT mice. Muscle fibers from *Sod1*^*−/−*^ mice showed substantially reduced generation of superoxide in response to contractions compared with fibers from WT mice. Inhibition of NOS did not affect DHE oxidation in fibers from WT or *Sod1*^*−/−*^ mice at rest or during contractions, but transgenic mice overexpressing nNOS showed increased DAF-FM fluorescence and reduced DHE oxidation in resting muscle fibers. It is concluded that formation of peroxynitrite in muscle fibers is a major effect of lack of SOD1 in *Sod1*^*−/−*^ mice and may contribute to fiber loss in this model, and that NO regulates superoxide availability and peroxynitrite formation in muscle.

## Introduction

The loss of muscle mass and strength that occurs during aging contributes to frailty and loss of independence (Marcell, 2003). By the age of 70, the cross-sectional area of skeletal muscle is reduced by 25–30%, and muscle strength is reduced by 30–40% (Porter *et al.*, 1995). Oxidative damage has been claimed to be involved in the loss of tissue function that occurs during aging, and skeletal muscle of old rodents contains increased amounts of the products of oxidative damage to biomolecules such as lipid, DNA, and proteins in comparison with young or adult rodents (e.g. Sastre *et al.*, 2003; Broome *et al.*, 2006; Vasilaki *et al.*, 2007). An increase in superoxide generation has been implicated in the process of aging in skeletal muscle and other tissues (Melov *et al.*, 2000; Sastre *et al.*, 2003). Mice lacking Cu,Zn superoxide dismutase (SOD1) show an accelerated, age-related loss of skeletal muscle mass associated with significant changes in muscle structure and contractility and potentially provide a useful model to study the role of a chronic oxidative stress in loss of skeletal muscle (Muller *et al.*, 2006).

Superoxide and nitric oxide (NO) are the primary radical species generated by skeletal muscle, and their activities increase during contractile activity (Reid *et al.*, 1992; Balon & Nadler, 1994; Kobzik *et al.*, 1994; Close *et al.*, 2005). Superoxide and NO are the precursors for the generation of a number of secondary species and muscle, and other tissues have sophisticated enzymatic systems that control the cellular activities of these species. Intracellular superoxide is regulated by the activities of the superoxide dismutases, SOD1 (Cu,ZnSOD, located in the cytosol and mitochondrial inter-membrane space) and SOD2 (MnSOD, located in the mitochondrial matrix). When superoxide and NO are both present, their chemical reaction to form peroxynitrite is likely and competes with the dismutation of superoxide to hydrogen peroxide by SOD (Beckman & Koppenol, 1996).

In mice lacking SOD1, the loss of muscle fibers that contributes to the accelerated muscle aging phenotype may therefore be associated with excess superoxide within the muscle cells, but it is possible that alternative species play important roles in the initiation of degeneration. Thus, peroxynitrite or a change in NO bioavailability because of reaction with excess superoxide might both affect tissue function. In recent studies, we have demonstrated that muscle fibers from SOD1 null mice show an increase in oxidation of the nonspecific intracellular reactive oxygen species (ROS) probe, 2′,7′-dichlorodihydrofluorescin-diacetate (DCFH), at rest compared with fibers from wild-type (WT) mice (Vasilaki *et al.*, 2010), but surprisingly show no further increase in oxidation of the probe following contractile activity, whereas an increase in DCFH oxidation was seen in fibers from WT mice following contractile activity. DCFH is reported to be relatively insensitive to oxidation by superoxide, but to be oxidized by hydrogen peroxide, hydroxyl radicals, peroxynitrite, and NO (Murrant & Reid, 2001).

Monitoring of the amounts or activities of specific ROS in cells is inherently difficult because of the labile and reactive nature of these species. The current study used a published technique for examination of intracellular NO in single muscle fibers (Pye *et al.*, 2007), but no technique for direct monitoring of peroxynitrite formation in muscle cells is available. Evidence for peroxynitrite formation has previously been implied from changes in the content of 3-nitrotyrosines (3-NT) in muscle proteins and from changes in superoxide and/or NO activities (Beckman & Koppenol, 1996; Pattwell *et al.*, 2004; Close *et al.*, 2005; Vasilaki *et al.*, 2006). Oxidation of DHE was used as a means of monitoring superoxide by following ethidium and 2-hydroxyethidium (2-HE) formation. This technique has only been sparsely used to examine skeletal muscle (Zuo *et al.*, 2000, 2004), and hence, an initial objective was to examine the reliability of this approach as a means of monitoring intracellular superoxide activities in muscle fibers.

The aims of the study were therefore to: (i) determine whether a lack of SOD1 in muscle was associated with an increase in superoxide availability, (ii) determine whether a lack of SOD1 in muscle was associated with a change in NO availability or an increase in peroxynitrite reactions, (iii) determine whether the lack of SOD1 in muscle was associated with changes in NOS enzymes, and (iv) determine whether the experimental manipulation of intracellular NO availability might modify superoxide availability in muscle cells. Our hypothesis was that when enzymatic dismutation of superoxide in muscle cytosol was absent because of lack of SOD1, the muscle would have increased intracellular superoxide activity, decreased intracellular NO content, and increased formation of 3-nitrotyrosine groups on muscle proteins because of increased formation of peroxynitrite. It was also hypothesized that manipulation of intracellular NO through inhibition of NOS enzymes or transgenic overexpression of nNOS would have little effect on muscle superoxide activities because NO is inevitably present in considerable excess of superoxide in muscle fibers.

## Results

### DHE oxidation by single muscle fibers from *Sod1*^*−/−*^ mice

**Figure 1:**
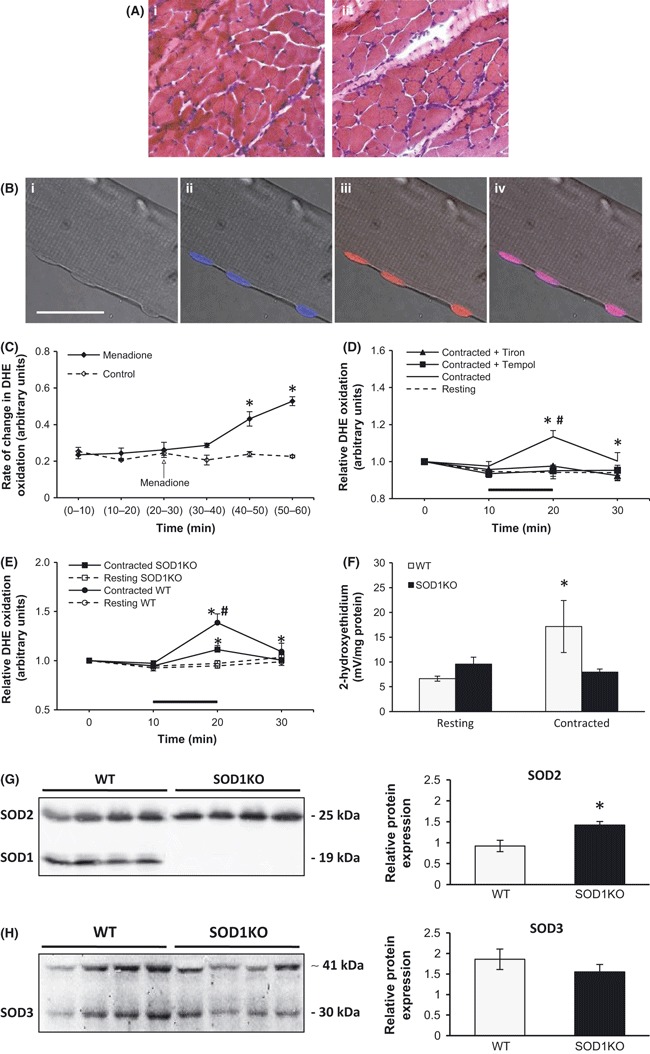
(A) Example of hematoxylin and eosin stained 6 μm transverse sections of the *flexor digitorum brevis* (FDB) muscle from wild-type (WT) (i) and *Sod1*^*−/−*^ (ii) mice. (B) Single isolated fiber from the FDB muscle after 24 h in culture photographed under bright field (bar = 30 μm) (i), fluorescent image following loading with 4′,6-diamidino-2-phenylindole dihydrochloride (DAPI) (ii) fluorescent image following loading with dihydroethidium (DHE) (iii) and a merged image of ii and iii (iv). (C) Rate of change in ethidium fluorescence from DHE-loaded FDB fibers either untreated or treated with menadione at 30 min. *P < 0.05 compared with values for untreated fibers at the same time point (*n* = 5–7 fibers in each group). (D) Relative ethidium fluorescence from FDB fibers over 30 min at rest, following 10 min of electrically stimulated contractions between 10 and 20 min with no treatment, and from fibers stimulated to contract between 10 and 20 min following pretreatment with the superoxide scavengers Tiron or Tempol. *P < 0.05 compared with fibers from the same group at the previous time point; ^#^*P* < 0.05 compared with fibers at rest or contracted fibers treated with Tiron or Tempol at the same time point (*n* = 7 fibers in each group). (E) Relative ethidium fluorescence from DHE-loaded fibers of WT and *Sod1*^*−/−*^ mice over 30 min at rest and following 10 min of electrically stimulated contractions between 10 and 20 min. *P < 0.05 compared with values for fibers from the same group at the previous time point; ^#^*P* < 0.05 compared with stimulated fibers from *Sod1*^*−/−*^ mice at the same time point (*n* = 7–8 mice in each group). (F) 2-hydroxyethidium content of fibers from WT and *Sod1*^*−/−*^ mice at rest and following 10 min of electrically stimulated contractile activity. *P < 0.05 compared with values for resting fibers from WT mice (*n* = 4 mice in each group). (G) Representative western blots of SOD1 and SOD2 proteins in *gastrocnemius* muscles of WT and *Sod1*^*−/−*^ mice and densitometric quantification of the blots for SOD2, *P < 0.05 compared to values for muscles from WT mice. (H) Representative western blots of SOD3 proteins in *gastrocnemius* muscles of WT and *Sod1*^*−/−*^ mice and densitometric quantification of the SOD3 band (see text for explanation of the presence of two bands).

To examine ROS activities in muscle fibers without any influence from nonmyogenic cells, single fibers from the *flexor digitorum brevis* (FDB) muscle were examined. Transverse sections of the FDB muscles from WT and *Sod1*^*−/−*^ mice stained with Hematoxylin and Eosin are shown in Fig. 1A. The FDB from *Sod1*^−/−^ mice showed minor changes in structure, such as greater variability in fiber size and the presence of occasional central nuclei compared with WT mice. Isolated fibers from the FDB were loaded with dihydroethidium (DHE), and images of a single isolated fiber from an adult WT mouse are shown in Fig. 1B as a bright field image (i), fluorescent image from the fiber labeled with 4′,6-diamidino-2-phenylindole dihydrochloride (DAPI) to visualize nuclei (ii), fluorescence image from the DHE-loaded fiber (iii), and merge of the images to show the co-localization of DAPI and ethidium fluorescence (iv). The predominant ethidium fluorescence from the DHE-loaded fiber was from nuclei although smaller amounts of fluorescence can be seen in areas of the fiber where nuclei were not apparent (Fig. 1B iii). In preliminary experiments, fluorescence measurements were undertaken from nuclei, non-nuclear areas of the fiber, and the whole fiber and the fluorescence values compared. These data (not shown in detail) indicated that the pattern of change of fluorescence from each of these measurements was very similar regardless of where the analyses were undertaken although the absolute fluorescence values differed. The ethidium generated by oxidation of DHE binds to nucleic acids and therefore effectively accumulates in nuclei. The fluorescence observed in nuclei therefore reflects overall oxidation in the muscle fiber rather than in nuclei specifically. Measurements from nuclei were therefore used for all further studies, because fluorescence values were much higher from this site, and hence, the sensitivity of the method might be optimized.

The rate of change in ethidium fluorescence from nuclei of resting untreated FDB fibers over 60 min is shown in Fig. 1C, and this figure also shows the effect of addition of menadione (5 μm) after 30 min. Menadione provides a positive control for increased superoxide generation and caused an increase in DHE oxidation that was significantly higher than in untreated fibers within 20 min.

Figure 1D shows the net effect of 10-min electrical stimulation of contractions on the changes in DHE oxidation in FDB fibers in the absence and presence of the superoxide scavengers, Tiron or Tempol. Ethidium fluorescence was significantly increased by the contraction protocol, but this increase was abolished in the presence of either compound. In contrast, neither Tempol nor Tiron had any significant effect on ethidium fluorescence from fibers at rest.

Ethidium fluorescence from nuclei of FDB fibers from WT and *Sod1*^*−/−*^ mice at rest and following a 30-min period of contractions is shown in Fig. 1E. These data showed that the fluorescence from fibers of the two groups did not differ at rest although the mean values from fibers of the *Sod1*^*−/−*^ mice tended to be higher. The effect of 10 min of electrically stimulated contractions on ethidium fluorescence from FDB fibers of WT and *Sod1*^*−/−*^ mice is also shown in Fig. 1E. Following contractile activity, a significant increase in fluorescence was seen from muscle fibers of WT mice. Fibers from the *Sod1*^*−/−*^ mice also showed a significant increase with contractions, but the overall increase was much smaller and significantly less than that seen from the fibers of WT mice.

Because there has been considerable recent debate about the validity of monitoring of ethidium formation from DHE as a means of assessing intracellular superoxide, the formation of 2-HE within fibers was also determined using high performance liquid chromatography (HPLC) techniques. These data are shown in Fig. 1F and also show no significant change in 2-HE formation by quiescent fibers from *Sod1*^*−/−*^ mice compared with those from WT mice. Following the 10-min contraction protocol, the 2-HE content of fibers from WT mice was significantly increased, but there was no effect of contractions on the 2-HE content of fibers from the *Sod1*^*−/−*^ mice.

The possibility that the lack of SOD1 might have led to adaptive changes in the contents of other SOD enzymes was examined by analysis of *gastrocnemius* muscles. Figure 1G,H show representative western blots from the muscles of WT and *Sod1*^***−/−***^ mice together with quantification of these blots by densitometry. As anticipated, SOD1 was absent from the muscles of *Sod1*^*−/−*^ mice, but there was a small but significant increase in the content of SOD2 (Fig. 1G). The contents of extracellular SOD (SOD3) were unchanged in muscles of *Sod1*^*−/−*^ mice compared with WT mice (Fig. 1H).

### NO and peroxynitrite in muscles of *Sod1*^*−/−*^ mice

**Figure 2:**
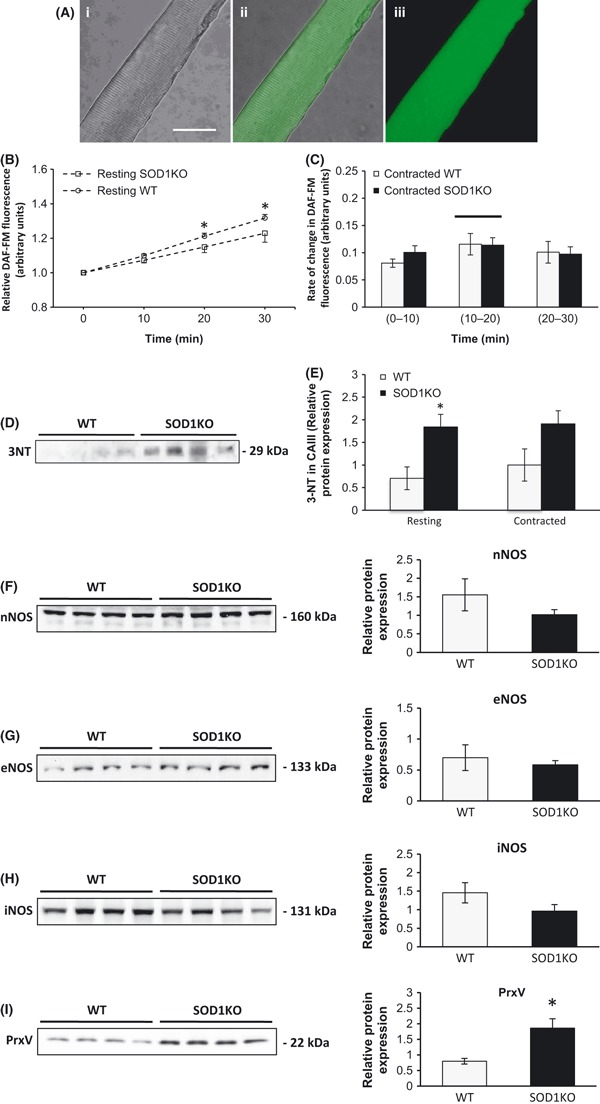
(A) Image of single isolated fiber from the *flexor digitorum brevis* (FDB) muscle after 24 h in culture under bright field (bar = 30 μm) (i), fluorescent image following loading with 4-amino-5-methylamino-2′,7′-difluorofluorescein diacetate (DAF-FM) (iii) and merge of i and iii (ii). (B) Relative DAF-FM fluorescence from DAF-FM DA-loaded fibers of wild-type (WT) and *Sod1*^*−/−*^ mice over 30 min at rest. *P < 0.05 compared with values for fibers from WT mice at the same time point (*n* = 6–7 mice in each group). (C). Rate of change in DAF-FM fluorescence from fibers from FDB muscles of WT and *Sod1*^*−/−*^ mice over 30 min with 10 min of electrically stimulated contractions between 10 and 20 min (*n* = 5–11 mice in each group). (D) Representative western blots of the 3-nitrotyrosine (3-NT) content of CAIII in *gastrocnemius* muscles of WT and *Sod1*^*−/−*^ mice at rest. (E) 3-NT content of CAIII in gastrocnemius muscles of WT and *Sod1*^*−/−*^ mice both at rest and following 15 min of isometric contractions *in vivo*. *P < 0.05 compared with values for resting muscles of WT mice. (F) Representative western blots of nNOS protein in *gastrocnemius* muscles of WT and *Sod1*^*−/−*^ mice and densitometric quantification of the blots. (G) Representative western blots of eNOS protein in *gastrocnemius* muscles of WT and *Sod1*^*−/−*^ mice and densitometric quantification of the blots. (H) Representative western blots of iNOS protein in *gastrocnemius* muscles of WT and *Sod1*^*−/−*^ mice and densitometric quantification of the blots. (I) Representative western blots of peroxiredoxin V (PrxV) protein in *gastrocnemius* muscles of WT and *Sod1*^*−/−*^ mice and densitometric quantification of the blots. *P < 0.05 compared with values for resting muscles of WT mice.

The effect of a lack of SOD1 on NO and peroxynitrite was assessed by monitoring DAF-FM fluorescence from muscle fibers of WT and *Sod1*^*−/−*^ mice as a measure of NO availability and the 3-nitrotyrosine content of the major muscle protein carbonic anhydrase III (CAIII) as a means of assessing the extent of peroxynitrite reactions. Images of the DAF-FM fluorescence from a resting fiber from WT mice are shown in Fig. 2A and indicate a lack of any sub-cellular localization of this fluorescent probe. The DAF-FM fluorescence from fibers of WT and *Sod1*^*−/−*^ mice at rest is shown in Fig. 2B. Fibers from WT mice showed a significant increase in DAF-FM fluorescence over time at rest, but this rate of increase was significantly lower in fibers from *Sod1*^*−/−*^ mice (Fig. 2B). Fibers from WT and *Sod1*^*−/−*^ mice showed a tendency to increase the rate of DAF-FM fluorescence following contractions, but this did not reach statistical significance for either group (Fig. 2C).

An example western blot of the 3-NT content of the major muscle protein, CAIII, in *gastrocnemius* muscles from the WT and *Sod1*^*−/−*^ mice is shown in Fig. 2D. Quantification of the 3-NT blots is shown in Fig. 2E. *Sod1*^*−/−*^ mice showed a significant increase in the 3-NT content of CAIII in comparison with muscle from WT mice at rest. Following electrical stimulation of contractions *in vivo*, the 3-NT content of the protein in muscle from WT mice tended to increase, but this was not statistically significant. No further increase in 3-NT content was seen from muscles of the *Sod1*^*−/−*^ mice following contractile activity (Fig. 2E).

The possibility that the lack of SOD1 might have induced adaptations in the content of NOS enzymes and thus influence NO availability was examined by western blot analysis of the *gastrocnemius* muscles from WT and *Sod1*^*−/−*^ mice. All of the three NOS isoforms (nNOS, eNOS, and iNOS) were detected in muscles from WT and *Sod1*^***−/−***^ mice, but no significant differences in contents were seen (Fig. 2F–H). In light of the increase in 3-NT content of muscle CAIII observed in *Sod1*^*−/−*^ mice, western blots were also probed for peroxiredoxin V (PrxV) because this protein is a peroxynitrite reductase and known to be increased in conditions where peroxynitrite is increased. A typical western blot together with quantification of the PrxV bands is shown in Fig. 2I. These indicate a significant increase in the PrxV content of *gastrocnemius* muscles from *Sod1*^*−/−*^ mice compared with WT mice.

### Effect of modification of NO availability on DHE oxidation in muscles of wild-type and *Sod1*^*−/−*^ mice

#### Effect of NOS inhibition

**Figure 3:**
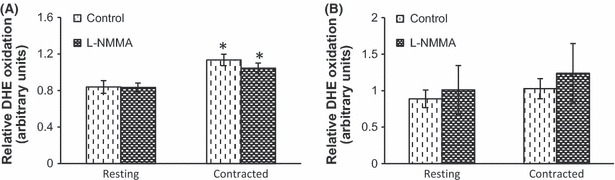
(A) Relative change in ethidium fluorescence from *flexor digitorum brevis* (FDB) fibers of wild-type (WT) mice prior to, and following 10 min of electrically stimulated contractions. Fibers were either untreated or treated with the nitric oxide synthases (NOS) inhibitor *N*^G^-monomethyl-l-arginine (l-NMMA). *P < 0.05 compared with fibers from WT mice at rest (*n* = 4 mice in each group). (B) Relative change in ethidium fluorescence from FDB fibers of *Sod1*^*−/−*^ mice prior to, and following 10 min of electrically stimulated contractions. Fibers were either untreated or treated with the NOS inhibitor l-NMMA (*n* = 4 mice in each group).

In previous studies, treatment with the NOS inhibitor *N*^G^-monomethyl-l-arginine (l-NMMA) was shown to inhibit NO generation by skeletal muscle in response to contractile activity (Pye *et al.*, 2007) with no effect on basal DAF-FM fluorescence. Fibers from WT mice treated with l-NMMA showed no differences in the rate of DHE oxidation compared with fibers from WT mice either at rest, or following contractile activity. Both the l-NMMA-treated and untreated fibers showed a significant increase in DHE oxidation following contractions (Fig. 3A). l-NMMA treatment of fibers from the *Sod1*^*−/−*^ mice also had no effect on DHE oxidation either at rest or following contractile activity compared with untreated fibers, although in fibers from the *Sod1*^*−/−*^ mice, no significant increase was seen following contractions (Fig. 3B).

#### Effect of nNOS overexpression

**Figure 4:**
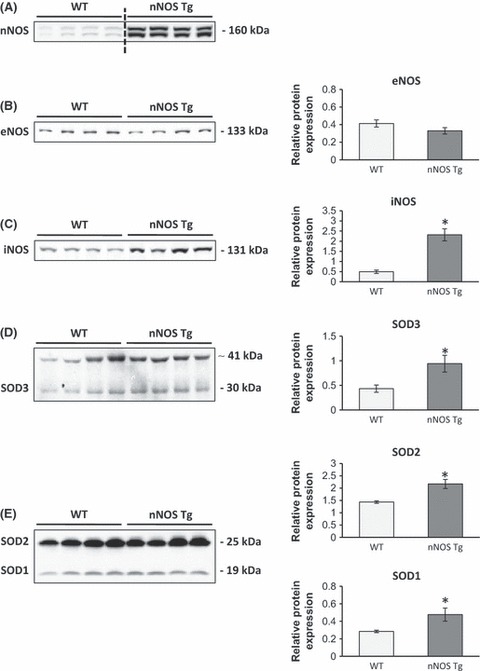
Representative western blots of protein from *gastrocnemius* muscles of wild-type (WT) and *nNOS*^*Tg*^ mice and densitometric quantification of the blots for nNOS (A), eNOS (B), iNOS (C), SOD3 (D) and SOD1 and 2 (E). *P < 0.05 compared with values for muscles of WT mice (*n* = 4 mice in each group). Because of the large difference in content of the nNOS protein in *nNOS*^*Tg*^ mice compared with WT mice, densitometry data are not presented for this protein. To produce the example blot for nNOS (A), samples from the muscles of *nNOS*^*Tg*^ mice were exposed in the Biorad Chemi-Doc System for 10 s compared with WT mice, whereas a 10-min exposure was required to demonstrate the low endogenous content of the protein, and hence, direct comparisons are not possible.

The data obtained from studies of single fibers from *Sod1*^*−/−*^ and WT mice indicated that any increased superoxide in the fibers from *Sod1*^*−/−*^ mice had reacted rapidly with NO to generate peroxynitrite. To examine whether an increase in NO production could decrease superoxide availability and DHE oxidation in muscle through increasing the formation of peroxynitrite, mice overexpressing nNOS (*nNOS*^*Tg*^ mice) were examined. Western blots of the muscle content of the three NOS isoenzymes (nNOS, eNOS, and iNOS) and the three SOD enzymes (SOD1, SOD2, and SOD3) in the muscles of *nNOS*^*Tg*^ mice in comparison with WT are shown in Fig. 4A–E. *nNOS*^*Tg*^ mice showed a > 100-fold increase in nNOS content with the western blot showing the two nNOS bands (full-length nNOS and a nNOS fragment) described by Nguyen & Tidball (2003). Muscles of *nNOS*^*Tg*^ mice also showed a significant increase in iNOS content compared with WT mice and increased content of all three SOD enzymes (Fig. 4C–E). The content of eNOS in muscles from *nNOS*^*Tg*^ mice was unchanged compared with that seen in WT mice (Fig. 4B).

**Figure 5:**
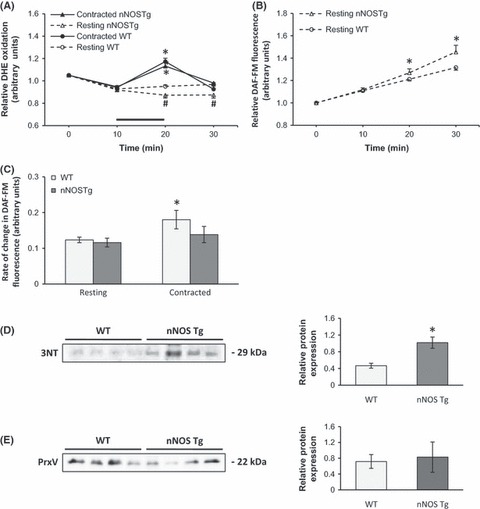
(A) Relative ethidium fluorescence from dihydroethidium (DHE)-loaded *flexor digitorum brevis* (FDB) fibers of wild-type (WT) and *nNOS*^*Tg*^ mice over 30 min. Fibers were either untreated or subjected to 10 min of electrically stimulated contractions between 10 and 20 min. *P < 0.05 compared with values at the previous time point for fibers from the same group; ^#^*P* < 0.05 compared to resting fibers from WT mice (*n* = 7–8 mice in each group). (B) Relative 4-amino-5-methylamino-2′,7′-difluorofluorescein (DAF-FM) fluorescence from resting fibers of WT and *nNOS*^*Tg*^ mice over 30 min. *P < 0.05 compared with fibers from WT mice at the same time point (*n* = 7–8 mice in each group). (C) Rate of change in DAF-FM fluorescence from fibers of WT and *nNOS*^*Tg*^ mice prior to, and following 10 min of electrically stimulated contractions. *P < 0.05 compared to fibers from WT mice prior to contractions (*n* = 7–8 mice in each group). (D) Representative western blots of the 3-nitrotyrosine content of CAIII in *gastrocnemius* muscles of WT and *nNOS*^*Tg*^ mice at rest and densitometric quantification of the blots. *P < 0.05 compared with values for muscles from WT mice (*n* = 7–8 mice in each group). (E) Representative western blots of peroxiredoxin V (PrxV) protein in *gastrocnemius* muscles of WT and *nNOS*^*Tg*^ mice and densitometric quantification of the blots.

The effect of nNOS overexpression on DHE oxidation in single muscle fibers is shown in Fig. 5A. This shows a small but significant decrease in DHE oxidation in resting fibers from the *nNOS*^*Tg*^ mice compared with WT, but no significant difference between the groups was seen following contractile activity (Fig. 5A). DAF-FM fluorescence from resting fibers of *nNOS*^*Tg*^ and WT mice is shown in Fig. 5B and demonstrates that the fibers from *nNOS*^*Tg*^ mice had increased DAF-FM fluorescence. Following contractile activity, a significant increase in DAF-FM fluorescence was seen from fibers of WT mice, but no significant increase was seen from fibers of the nNOS transgenic mice (Fig. 5C). The 3-NT content of CAIII in the *gastrocnemius* muscles of WT and *nNOS*^*Tg*^ mice is shown in Fig. 5D and shows a significant increase in 3-NT formation in the muscle of the *nNOS*^*Tg*^ mice. The PrxV content of the *gastrocnemius* muscles from WT and *nNOS*^*Tg*^ mice is shown in Fig. 5E. No differences in PrxV content were seen.

## Discussion

Superoxide and NO are the primary reactive oxygen and nitrogen species generated within skeletal muscle both at rest and during contractile activity. While further reaction of both species is well described with the generation of secondary ROS such as hydrogen peroxide and hydroxyl radicals from superoxide (Jackson, 2008) and interaction of NO with targets such as guanylate cyclase (Halliwell & Gutteridge, 2007), the reaction of superoxide with NO has been described in simple chemical and some biological systems (Beckman & Koppenol, 1996), but the functional effects of this interaction in skeletal muscle have not been defined.

In nonstimulated cells, NO is present in the cytoplasm at 10^−9^ to 10^−8^ m, whereas superoxide is reported to be present at 10^−12^ to 10^−13^ m (Chance *et al.*, 1979; Halliwell & Gutteridge, 2007). The chemical reaction of superoxide with NO to generate peroxynitrite has a reaction rate of ∼7 × 10^9^ m^−1^ s^−1^ which is approximately threefold higher than the superoxide dismutase catalyzed conversion of superoxide to hydrogen peroxide of ∼2 × 10^9^ m^−1^ s^−1^ (Beckman & Koppenol, 1996). Thus, theoretically, an increase in superoxide generation will lead to peroxynitrite formation in preference to formation of hydrogen peroxide in muscle and other cells. NO is usually present in cells to at least 100-fold excess, but the relatively large quantity of superoxide dismutase present in cytoplasm (SOD1) or mitochondria (SOD2) of cells appears to ensure that hydrogen peroxide is a major product under normal physiological conditions. Where superoxide generation or flux is increased, it is unclear what proportion of the extra superoxide might generate peroxynitrite in the presence of excess NO. Such a situation may be present in aging where increased generation of superoxide has been implicated in the loss of muscle mass and function that occurs (Melov *et al.*, 2000; Sastre *et al.*, 2003). Data from Muller *et al.* (2006) indicated that mice lacking Cu,ZnSOD (SOD1) showed an accelerated skeletal muscle aging phenotype that is characterized by loss of muscle fibers and degeneration of the innervating motor neurons (Kostrominova *et al.*, 2007). In addition, adult mice lacking SOD1 also mimic other aspects of the normal aging phenotype such as an inability to activate adaptive responses to contractions (Vasilaki *et al.*, 2010). There is therefore considerable interest in establishing the changes in ROS and NO that occur in *Sod1*^*−/−*^ mice because this may inform further mechanistic studies during normal aging.

Mice lacking SOD1 showed a small but significant increase on muscle SOD2 content that may reflect the increased number of mitochondria reported to be present in muscles of these mice (Kostrominova *et al.*, 2007). No significant changes in the muscle contents of SOD3 or of the three NOS isoforms were seen. Larger limb muscles of the *Sod1*^*−/−*^ mice, such as the *gastrocnemius*, have been shown to have a decreased number of fibers (Muller *et al.*, 2006) compared with WT mice, but although the FDB has similar fiber-type composition and would be anticipated to show analogous production of ROS etc., the FDB muscle showed only minor changes in structure compared with WT. An increase in the variability of fiber sizes and numbers of fibers having central nuclei is apparent which appear to reflect previous cycles of degeneration and regeneration (Fig. 1A).

### DHE oxidation by single muscle fibers from *Sod1*^*−/−*^ mice

Initial studies examined the sensitivity of monitoring DHE oxidation as a means of assessing superoxide activity in muscle fibers. The single isolated mature skeletal muscle fiber preparation from the mouse FDB muscle has been previously used with ROS-sensitive fluorescent probes (Pye *et al.*, 2007; Palomero *et al.*, 2008; Vasilaki *et al.*, 2010) and was applied here. Cell permeable DHE reacts with superoxide to form cell impermeable ethidium which binds to nucleic acids that are predominantly within nuclei (Benov *et al.*, 1998; Tarpey *et al.*, 2004; Zuo *et al.*, 2004). Most studies in nonmuscle cells have examined whole cell fluorescence of ethidium, but because skeletal muscle fibers are multi-nuclear, the rapid accumulation of the ethidium in individual nuclei is clearly apparent (Fig. 2B). Measurements from nuclei were used to ensure optimal sensitivity of the technique. Exposure of DHE-loaded fibers to menadione, a known inducer of intracellular superoxide generation, caused the anticipated increase in ethidium fluorescence (Fig. 1C). An increase was also seen following 10 min of electrically stimulated contractions (Fig. 1D). This increase with contractile activity was abolished when fibers were preincubated with the superoxide scavengers Tiron or Tempol (Fig. 1D). Thus, the technique based on use of single mature skeletal muscle fibers loaded with DHE appeared capable of detecting cellular changes in superoxide that occur in response to the physiological stimulus of contractile activity.

Resting fibers from *Sod1*^*−/−*^ mice showed no increase in DHE oxidation compared with those from WT mice with no statistical differences between the groups over 30 min (Fig. 1E). In fibers from WT mice, contractile activity induced a substantial increase in ethidium fluorescence from DHE-loaded fibers, indicating an increase in superoxide reactions in response to contractions, but in muscle fibers from the *Sod1*^*−/−*^ mice, the contraction-induced increase in ethidium fluorescence was much smaller (Fig. 1E).

Thus, these data indicate that any rise in superoxide activity in fibers from the *Sod1*^*−/−*^*mice* at rest was much less than that which occurs during physiological contractions of skeletal muscle, and that the *Sod1*^*−/−*^*mice* generated much less superoxide in muscle than WT mice following contractions. These data were unexpected, and hence, the formation of 2-HE was also examined in wells of fibers from the *Sod1*^*−/−*^ and WT mice at rest. Although many studies have monitored ethidium fluorescence as an index of superoxide activity (e.g. Andreas *et al.*, 2000; Carriedo *et al.*, 1998; Dikalov *et al.*, 2007), recent studies have identified 2-HE as a specific product of the reaction of DHE with superoxide (Fink *et al.*, 2004). The 2-HE content of fibers measured by HPLC also showed no significant increase in the resting fibers of *Sod1*^*−/−*^ mice compared with those from WT mice confirming the apparent lack of any increase in superoxide availability in muscle fibers of the *Sod1*^*−/−*^ mice at rest. The 2-HE content of fibers from WT mice also increased following contractions, but no significant changes were induced by contractions in the fibers of *Sod1*^*−/−*^ mice.

### NO and peroxynitrite in muscles of *Sod1*^*−/−*^ mice

DAF-FM fluorescence from fibers of *Sod1*^*−/−*^ mice at rest was decreased to a small but significant extent throughout the period of study compared with that from fibers of WT mice (Fig. 2B). These data are compatible with a reduced NO availability in fibers from the *Sod1*^*−/−*^ mice. Following contractions, the fibers from both WT and *Sod1*^*−/−*^ mice showed a tendency to an increase in DAF-FM fluorescence, but this did not reach statistical significance. The 3-NT content of CAIII in muscles was also increased in fibers from *Sod1*^*−/−*^ mice compared with those from WT mice at rest with no further increase following contractions. These data are therefore entirely compatible with an increased generation of peroxynitrite in the muscle fibers of *Sod1*^*−/−*^ mice at rest that does not further increase during contractions. Previous studies from this laboratory (Vasilaki *et al.*, 2006) have indicated that measurements of the 3-NT content of the major cytosolic protein CAIII are a more sensitive marker of oxidative modifications to muscle than measurements of total muscle 3-NT content and also indicated that the 3-NT content of muscle CAIII showed a small but significant increase following contractile activity. A tendency to an increase was seen for WT mice in the current study, but the overall increase in muscle 3-NT content was much larger in the *Sod1*^*−/−*^ mice at rest than was induced by contractions in the muscles of WT mice. These data contrast with the DHE oxidation results which indicated that the changes in muscle superoxide availability were much less in the *Sod1*^*−/−*^ mice at rest than the changes induced by contractions in the muscles of WT mice. The western blotting approach used to assess the 3-NT content of muscle proteins in the current study is not sufficiently sensitive to demonstrate any potential changes in the 3-NT content of relatively low abundance proteins in the cytosol or mitochondrial intermembrane space of muscle (Vasilaki *et al.*, 2006) although such analyses might provide information on important sites for peroxynitrite action in the *Sod1*^*−/−*^ mice.

All of the six mammalian Prx proteins act to degrade hydrogen peroxide (Wood *et al.*, 2003), but PrxV also has the highest reported peroxynitrite reductase activity (Dubuisson *et al.*, 2004; Trujillo *et al.*, 2008). This function appears important *in vivo* because previous studies have shown that changes in PrxV expression modulated peroxynitrite toxicity, and the cellular content of the enzyme was found to be upregulated in conditions associated with increased peroxynitrite formation (Trujillo *et al.*, 2008). The muscle content of PrxV was found to be increased in *Sod1*^*−/−*^ mice compared with WT mice which provides further support for a substantial increase in peroxynitrite generation in this model.

### Can modification of NO availability affect superoxide availability (DHE oxidation) in muscles of wild-type and *Sod1*^*−/−*^ mice?

We have previously shown that the NOS inhibitor, l-NMMA, abolished the contraction-induced increase in DAF-FM fluorescence in isolated FDB fibers from WT mice (Pye *et al.*, 2007), but in the current study, it had no significant effect on ethidium fluorescence from DHE-loaded fibers of either the SOD1 knockout or WT mice. Thus, we conclude that even where any additional NO generated by contractions was abolished, no increase in superoxide was detected during contractions in muscle fibers from *Sod1*^*−/−*^ mice. This lack of effect of the NOS inhibitor on superoxide availability at rest may relate to the ∼100-fold excess of NO over superoxide that is present in tissues (Chance *et al.*, 1979; Halliwell & Gutteridge, 2007). In our previous study (Pye *et al.*, 2007), the NOS inhibitor, l-NMMA, had no effect on the DAF-FM fluorescence from resting fibers despite abolishing the increase seen with contractions and we argued that this was because of the presence of other sources for NO in muscle cells including S-nitrosothiols (Bryan *et al.*, 2004; Rayner *et al.*, 2005). Hence, inhibition of NOS may not decrease cytosolic NO to a sufficient extent to remove the excess available for reaction with superoxide and formation of peroxynitrite.

To determine whether a substantial increase in NO formation might reduce superoxide bioavailability in muscle fibers of WT mice, the effect of overexpression of nNOS in muscle was also examined. Initial studies demonstrated the substantial increase in nNOS content that occurred in the muscle of *nNOS*^*Tg*^ mice and also, surprisingly, that this was associated with an increase in iNOS content (Fig. 4C). All three of the SOD isoforms were found to be elevated in the muscle of the *nNOS*^*Tg*^ mice compared with WT. These data indicate a substantial adaptive response to the overexpression of nNOS in muscle and further demonstrate the close link between NO and superoxide metabolism in this tissue. Muscle fibers from *nNOS*^*Tg*^ mice had a significant increase in DAF-FM fluorescence at rest in comparison with those from WT mice, but showed no further increase with stimulation such that the rate of increase in DAF-FM fluorescence was equivalent between the fibers from nNOS and WT mice following contractile activity. These data indicate that nNOS plays a significant role on NO generation by muscle at rest as previously reported (Nguyen & Tidball, 2003). In contrast, they do not support the possibility that this source plays a significant role in the stimulation-induced additional release of NO, although further knowledge of the location and regulation of the transgenic nNOS compared with endogenous nNOS would be required to fully evaluate this possibility. The nNOS overexpression appeared to reduce the superoxide availability in fibers from WT mice at rest because DHE oxidation was reduced in fibers from the *nNOS*^*Tg*^ mice in comparison with WT mice. The possibility that this was because of increased reaction with superoxide to generate peroxynitrite is supported by the increase in the 3-NT content of CAIII in the muscle of *nNOS*^*Tg*^ mice compared with WT mice that was seen (Fig. 5D), although the adaptive changes in SOD contents seen in muscles of the *nNOS*^*Tg*^ mice (Fig. 4D,E) may also have played a role to reduce superoxide availability and hence DHE oxidation.

The overall increase in peroxynitrite in muscles of the *nNOS*^*Tg*^ mice appeared to be lower than that seen in the muscles of *Sod1*^*−/−*^ mice because the 3-NT content of CAIII was not increased to the same extent (Fig. 2D compared with Fig. 5D), and no upregulation of PrxV was seen in the *nNOS*^*Tg*^ mice, whereas the content of PrxV was increased in muscle of the *Sod1*^*−/−*^ mice. It is also likely that the predominant sub-cellular sites for increased peroxynitrite generation differed in the muscles of *Sod1*^*−/−*^ mice compared with *nNOS*^*Tg*^ mice because SOD1 is found in the cytoplasm and mitochondrial intermembrane space, whereas endogenous nNOS is localized to the inside of the muscle plasma membrane. PrxV is localized predominantly to mitochondria (Wood *et al.*, 2003), and hence, the increase in expression seen in muscle of *Sod1*^*−/−*^ mice supports a substantial increase in peroxynitrite in that compartment.

The western blots for SOD3 (extracellular SOD) in Figs 1H and 4D show two predominant bands. There is considerable discrepancy in the published literature concerning the pattern and molecular weight of SOD3 seen in mouse tissues. Published data show either a single band (e.g. Wiggers *et al.*, 2008), a doublet (e.g. see Kliment *et al.*, 2009) or the two major bands reported here (Lavina *et al.*, 2009). This pattern appears in part related to the nature of the antibody that was used, and detailed examination of the ∼30-kD band shown in Fig. 1H indicates that it is a doublet. The two bands in the doublet are thought to represent the ‘full-length’ and ‘proteolyzed’ forms of the protein in which the heparin-binding portion of the protein is removed. The higher molecular weight band in Figs 1H and 4D has been described as a highly glycosylated form of the protein (Lavina *et al.*, 2009).

In conclusion, these data indicate that removal of SOD-catalyzed conversion of cytosolic superoxide to hydrogen peroxide in *Sod1*^*−/−*^ mice caused no significant increase in the apparent superoxide availability in muscle fibers at rest, although a substantial increase in superoxide availability was observed following contractile activity in muscle fibers of WT mice. The lack of SOD1 caused a substantial increase in peroxynitrite formation which is likely to be due to the excess NO concentration that is present in the muscle fibers. Furthermore, the lack of SOD1 appeared to prevent the increase in generation of superoxide that occurs in muscle fibers during contractile activity in WT mice through an unexplained mechanism (Vasilaki *et al.*, 2010). The increase in basal peroxynitrite formation and decreased NO in muscles of *Sod1*^*−/−*^ mice was also associated with an accelerated age-related loss of muscle mass and function. An increase in superoxide activity has been implicated in the loss of skeletal muscle mass and function that accompanies aging, and several studies have attempted to modify superoxide in skeletal muscle through interventions to increase superoxide scavenging capacity without clear success (e.g. Melov *et al.*, 2000; Melov, 2002; Martin *et al.*, 2009). It is feasible that these studies may have been ineffective because of the inability of the additional superoxide scavengers to compete with NO for reaction with superoxide, and the data presented here indicate that alternative approaches based on scavenging of peroxynitrite may be more effective. Peroxynitrite has been shown to inactivate aconitase (Castro *et al.*, 1994; Hausladen & Fridovich, 1994) and is hypothesized to decompose to generate the hydroxyl radical which can oxidize proteins, lipid, and DNA and hence is a potential initiator of degradative processes in muscle (Beckman & Koppenol, 1996). In comparison to superoxide or hydroxyl radical, peroxynitrite is relatively stable, and therefore, there is the opportunity to scavenge this molecule prior to its decomposition although efficient approaches to this have not yet been described (Beckman & Koppenol, 1996; Guven *et al.*, 2008; Jiao *et al.*, 2009).

## Experimental procedures

### Mice and *in vivo* contractions of skeletal muscle

These studies used male adult (5–11 months) mice lacking SOD1 (C57Bl/6 *Sod1*^−/−^ mice) and C57Bl/6 WT mice and transgenic mice overexpressing nNOS (C57Bl/6 *nNOS*^*Tg*^ mice) and their control C57Bl/6 WT mice. Details of the generation and characterization of the knockout (Elchuri *et al.*, 2005; Muller *et al.*, 2006) and transgenic (Nguyen & Tidball, 2003) mouse models have been reported previously. Transgenic mice overexpressing the rat brain nNOS (type 1 NOS) in skeletal muscle were used (Wehling *et al.*, 2001). Expression of the nNOS transgene was driven by the human skeletal actin promoter and the vp1 intron. All experiments were performed in accordance with UK Home Office guidelines and under the UK Animals (Scientific Procedures) Act 1986. Mice were killed by an overdose of the anesthetic, and muscles were removed quickly. The FDB muscles were used for isolation of single fibers (see below), and other muscles were harvested and stored at −70°C until analysis.

In some studies, the muscles of one hind limb of mice were subjected to a protocol of isometric contractions *in vivo* as previously described (Vasilaki *et al.*, 2007). Adult WT and *Sod1*^*−/−*^ mice were anesthetized with pentobarbital sodium, with an initial dose of 65 mg per 100 g body mass given by intraperitoneal injection. Supplemental doses were administered as required to maintain a depth of anesthesia sufficient to prevent response to tactile stimuli. The knee of one hind limb was fixed to a base plate, and the hindlimb musculature was stimulated to contract by surface electrodes placed around the upper limb and the ankle to induce isometric contractions *in vivo* (McArdle *et al.*, 2001). Maximum isometric tetanic contractions were produced by square wave pulses of 0.2-ms duration, a voltage slightly greater than that required to produce a maximum twitch (usually ∼70 V), and a frequency of 100 Hz (McArdle *et al.*, 2001). Maximum isometric contractions were held for 500 ms, with a contraction every 4 s for a total of 225 contractions during the 15 min of the contraction protocol. Mice remained under anesthesia until the end of the contraction protocol. Mice were allowed to recover and killed by cervical dislocation at 15 min following the end of the contraction protocol. The *gastrocnemius* muscles were removed, weighed, and frozen in liquid nitrogen. Control, noncontracted muscles were obtained from WT and *Sod1*^−/−^ mice following sacrifice by an overdose of anesthetic.

### Western blotting of muscle proteins

For assessment of specific proteins in muscle, 100 μg of total protein was applied to a 8-15% polyacrylamide gel with a 4% stacking gel (National Diagnostics Ltd, Atlanta, Georgia, USA). The separated proteins were transferred onto nitrocellulose membranes by western blotting. The membranes were analyzed using antibodies to CuZnSOD (SOD1), MnSOD (SOD2) and extracellular SOD (SOD3) (Stressgen Inc., Victoria, BC, Canada), nNOS, eNOS and iNOS and PrxV (Abcam plc, Cambridge, UK) as previously described (McArdle *et al.*, 2001). The membranes were incubated with anti-mouse or anti-rabbit peroxidase-conjugated IgG antibodies where appropriate (Sigma Co., Dorset, UK). Peroxidase activity was detected using an enhanced chemiluminescence kit (Amersham International Cardiff, UK), and bands were visualized as described previously (McArdle *et al.*, 2001). All bands were identified in comparison with a sample that had not been exposed to the primary antibody and using molecular weight markers (Amersham International). All protein contents were normalized to the GAPDH content of the same sample. Comparisons were made between samples on the same gel/western blot.

### Analyses of the 3-nitrotyrosine content of muscle carbonic anhydrase III

Previous studies have indicated that the 3-nitrotyrosine (3-NT) content of the major muscle protein, carbonic anhydrase III, is a relatively sensitive marker of muscle oxidative stress (Vasilaki *et al.*, 2007), and this was examined using the techniques described by Vasilaki *et al.* (2007). Muscles were ground in a motor and pestle under liquid nitrogen and frozen muscle powder was placed into a buffer containing 1 mm iodoacetamide, 1 mm benzithonium chloride, 5.7 mm phenylmethylsulphonyl fluoride in 1% (w/v) SDS, homogenized on ice, and centrifuged at 10 000 *g* for 10 min at 4°C. Protein content of samples was determined using the bicinchoninic acid method. Total cellular protein (50 μg) was separated on 1D SDS-PAGE followed by western blotting. The content of 3-NT was analyzed by using a mouse monoclonal antibody (Cayman Chemical Co., Ann Arbor, Michigan, USA), and the bands were visualized using a Biorad Chemi-Doc System (Biorad Laboratories Ltd, Hemel Hempstead, UK). Densitometric quantification of the carbonic anhydrase III band was undertaken, and the protein content was normalized to the GAPDH content of the same sample. Comparisons were made between samples on the same gel/western blot.

### Isolation of single mature skeletal muscle fibers

Single fibers were isolated from the FDB muscle of mice according to the method of Shefer & Yablonka-Reuveni (2005). Briefly, mice were killed, and the FDB muscles were dissected. Muscles were incubated for 1.5 h at 37°C in 0.4% (w/v) Type I collagenase (EC 3.4.24.3; Sigma Chemical Co., Poole, Dorset, UK) in minimum essential medium eagle (MEM) containing 2 mm glutamine, 50 i.u. penicillin, 50 μg mL^−1^ streptomycin, and 10% fetal bovine serum (FBS). The muscles were agitated every 30 min during the digestion period. Single myofibers were released by gentle trituration with a wide-bore pipette, and fibers were washed three times in MEM containing 10% FBS. Fibers were plated onto precooled 35-mm cell culture dishes precoated with 50 μL of Matrigel (BD Biosciences) and were allowed to attach for 45 min before adding 1 mL MEM containing 10% FBS. Fibers were incubated for 16 h at 37°C in 5% CO_2_. Fibers prepared and cultured in this manner are viable for up to 6 days in culture (Palomero *et al.*, 2008). Analyses were only performed after 24 h in culture on fibers that displayed good morphology and prominent cross-striations.

### Monitoring of DHE oxidation to assess intracellular superoxide activity in isolated fibers

Fibers were loaded by incubation in 2 mL Dulbecco’s phosphate-buffered saline (D-PBS) containing 5 μm dihydroethidium (DHE) for 30 min at 37°C. Cells were then washed twice with D-PBS, and the fibers were maintained in MEM without Phenol Red during the experimental protocol. This method is based on that described by Zuo *et al.* (2000, 2004).

### Use of DAF-FM DA to monitor NO activity in isolated fibers

The method described by Pye *et al.* (2007) was used. Fibers were loaded by incubation in 2 mL D-PBS containing 10 μm 4-amino-5-methylamino-2′,7′-difluorofluorescein diacetate (DAF-FM DA; Molecular Probes, Eugene, OR, USA) for 30 min at 37°C. Cells were then washed twice with D-PBS, and the fibers were maintained in MEM without Phenol Red during the experimental period. DAF-FM DA readily diffuses into cells and within the cytoplasm releases DAF-FM by the action of intracellular esterases. DAF-FM is essentially nonfluorescent until it is nitrosylated by products of oxidation of NO, resulting in DAF-FM triazole that exhibits about a 160-fold greater fluorescence efficiency (Kojima *et al.*, 1999).

### Microscopy and fluorescent imaging

The image capture system consisted of a Zeiss Axiovert 200M microscope equipped with both 500/20 excitation, 535/30 emission and 510/60 excitation, 590 emission filter sets (Carl Zeiss GmbH, Welwyn Garden City, UK). Using a ×20 objective, fluorescence images were captured with a computer-controlled Zeiss MRm charged-coupled device (CCD) camera (Carl Zeiss GmbH) and analyzed with the Axiovision 4.0 image capture and analysis software (Carl Zeiss Vision GmbH).

### Analysis of 2-hydroxyethidium by HPLC

The 2-HE content of fibers was analyzed by HPLC as described by Zhao *et al.* (2005). All fibers in a 35-mm dish were loaded with 5 μm DHE for 30 min at 37°C and washed twice with D-PBS. At 15 min following loading, 2-HE was extracted by the addition of 0.5 mL *n*-butanol to the sample. Following vortex mixing, the butanol phase was separated and dried under nitrogen. The sample was reconstituted in 0.1 mL HPLC grade water prior to injection onto the HPLC system. Separation of 2-HE from ethidium was undertaken using a Ginkotech HPLC system with a C18 reverse phase column (Partisil ODS-3 250 × 4.5 mm). The solvent was initially 10% acetonitrile in 0.1% trifluoroacetic acid and increased linearly to 70% acetonitrile over 46 min with a flow rate of 0.5 mL min^−1^. Detection was by fluorescence with excitation at 510 and emission at 595 nm.

### Use of inhibitors/scavengers of superoxide or NOS enzymes

Incubation of fibers with scavengers of superoxide (Tiron, 4,5-dihydroxy-1,3-benzenedisulphonic acid, at 1 mm, or Tempol, 4-hydroxy-2, 2, 6, 6-tetramethylpiperidine 1-oxyl at 100 μm) or an inhibitor of NOS (l-NMMA at 1 mm) was commenced at 1 h prior to loading with DAF-FM DA or DHE, and the reagents were maintained in the incubation medium during the loading and experimental periods.

### Electrical stimulation of contractions by isolated fibers

Single muscle fibers were subjected to electrical field stimulation in 35-mm dishes using platinum electrodes as described by Pattwell *et al.* (2004) and McArdle *et al.* (2005). Following 10 min at rest, fibers were electrically stimulated with trains of bipolar square wave pulses of 2 ms in duration for 0.5 of a second repeated every 5 s at 50 Hz and 30 V per well.

### Statistical analyses

Data are presented as means ± SE for each experiment. Data were initially analyzed by analysis of variance followed by Tukey *post hoc* analysis. Data were considered significant at *P* < 0.05.
